# Health system barriers influencing timely breast cancer diagnosis and treatment among women in low and middle-income Asian countries: evidence from a mixed-methods systematic review

**DOI:** 10.1186/s12913-022-08927-x

**Published:** 2022-12-31

**Authors:** Agani Afaya, Sheena Ramazanu, Obasanjo Afolabi Bolarinwa, Vida Nyagre Yakong, Richard Adongo Afaya, Richard Gyan Aboagye, Silas Selorm Daniels-Donkor, Ahmed-Rufai Yahaya, Jinhee Shin, Veronica Millicent Dzomeku, Martin Amogre Ayanore, Robert Kaba Alhassan

**Affiliations:** 1 Mo-Im Nursing Research Institute, College of Nursing, Yonsei University, 50-1, Yonsei-ro, Seodaemun-gu, Seoul, 03722 Republic of Korea; 2 Department of Nursing, School of Nursing and Midwifery, University of Health and Allied Sciences, Ho, Ghana; 3 Alice Lee Centre for Nursing Studies, Yong Loo Lin School of Medicine, National University of Singapore, Singapore, Singapore; 4 Department of Global Public Health, Canterbury Christ Church University, Canterbury, UK; 5 Department of Preventive Health Nursing, School of Nursing and Midwifery, University for Development Studies, Tamale, Ghana; 6 Department of Midwifery and Women’s Health, School of Nursing and Midwifery, University for Development Studies, Tamale, Ghana; 7 Department of Family and Community Health, Fred N. Binka School of Public Health, University of Health and Allied Sciences, Hohoe, Ghana; 8 Department of Nursing, School of Health Sciences, University of Dundee, Scotland Dundee, UK; 9 Hariri School of Nursing, American University of Beruit, Beirut, Lebanon; 10 Department of Internal Medicine, Tamale Teaching Hospital, Tamale, Ghana; 11 Woosuk University, College of Nursing, Wanju, Republic of Korea; 12 Department of Nursing, College of Health Sciences, Kwame Nkrumah University of Science and Technology, Kumasi, Ghana; 13 Department of Health Policy Planning and Management, Fred N. Binka School of Public Health, University of Health and Allied Sciences, Ho, Ghana; 14 Centre for Health Policy and Implementation Research. Institute of Health Research, University of Health and Allied Sciences, Ho, Ghana

**Keywords:** Breast cancer, Health systems, Barriers, Asia

## Abstract

**Background:**

Globally, breast cancer is the most common cancer type and the leading cause of cancer mortality among women in developing countries. A high prevalence of late breast cancer diagnosis and treatment has been reported predominantly in Low- and Middle-Income Countries (LMICs), including those in Asia. Thus, this study utilized a mixed-methods systematic review to synthesize the health system barriers influencing timely breast cancer diagnosis and treatment among women in Asian countries.

**Methods:**

We systematically searched five electronic databases for studies published in English from 2012 to 2022 on health system barriers that influence timely breast cancer diagnosis and treatment among women in Asian countries. The review was conducted per the methodology for systematic reviews and reported following the Preferred Reporting Items for Systematic Reviews and Meta-Analyses (PRISMA) guidelines, while health system barriers were extracted and classified based on the World Health Organization (WHO)‘s Health Systems Framework. The mixed-methods appraisal tool was used to assess the methodological quality of the included studies.

**Results:**

Twenty-six studies were included in this review. Fifteen studies were quantitative, nine studies were qualitative, and two studies used a mixed-methods approach. These studies were conducted across ten countries in Asia. This review identified health systems barriers that influence timely breast cancer diagnosis and treatment. The factors were categorized under the following: (1) delivery of health services (2) health workforce (3) financing for health (4) health information system and (5) essential medicines and technology. Delivery of health care (low quality of health care) was the most occurring barrier followed by the health workforce (unavailability of physicians), whilst health information systems were identified as the least barrier.

**Conclusion:**

This study concluded that health system factors such as geographical accessibility to treatment, misdiagnosis, and long waiting times at health facilities were major barriers to early breast cancer diagnosis and treatment among Asian women in LMICs. Eliminating these barriers will require deliberate health system strengthening, such as improving training for the health workforce and establishing more healthcare facilities.

**Supplementary Information:**

The online version contains supplementary material available at 10.1186/s12913-022-08927-x.

## Background

Globally, breast cancer is the most commonly diagnosed cancer and the fifth cause of cancer deaths, with an estimated 2.3 million global prevalence and 685,000 deaths in 2020 [1, 2]. It is estimated that in 2070, the cases of breast cancer are expected to reach 4.4 million [2, 3]. Among women, breast cancer accounted for about 24.5% of all cancer cases and 15.5% of cancer mortality, ranking it as the number one for incidence and mortality in the majority of the world countries in 2020 [1, 2]. According to Global Cancer Statistics, breast cancer is the second most prevalent cancer in Asia, specifically among women [1]. Evidence shows that some Asian countries have had relatively low breast cancer incidence but have recently shown rapidly increasing trends due to social economic development and lifestyle changes [2, 4, 5].

In high-income countries, it is estimated that more than 70% of breast cancer patients are diagnosed in the early stages of cancer [stage 1 or 2] while in Low-and Middle-Income Countries (LMICs), about 20–50% are diagnosed with advanced breast cancers [6, 7]. Delayed presentation, diagnosis, and treatment of breast cancer are often associated with poor prognosis [8, 9] and account for the significant differences in the mortality rate of breast cancer in various countries [10]. Although breast cancer is a significant health problem in North America, Western Europe, and Australia [1], its incidence is currently observed to have significantly increased in several Asian nations [1]. For example, in Iran, the annual incidence of breast cancer is approximately 20 new cases per 100,000 women [11], out of which 70% are diagnosed at advanced cancer stages, leading to death within a short stipulated timeframe [12]. A delay in breast cancer diagnosis and treatment could be related to one of the following factors: patient screening delay, health or medical care provider delay, delay in services, or treatment delay [13]. Several underlying factors are associated with late diagnosis of breast cancer, including age, marital status, socioeconomic status, health insurance, history of benign breast disease, menopausal status, type of tumor, and type of first symptoms [7, 14–17].

A delay in cancer diagnosis does not only decrease patients’ chances of survival but may also increase medical costs requiring more invasive treatments [7]. Most of the existing review studies conducted in Asia were centered on factors associated with delayed presentation, diagnosis, and treatment of breast cancer [18, 19]. However, health system-related factors associated with the delayed diagnosis and treatment of breast cancer (from presentation to first treatment) have not been well investigated in the Asian context. A recent systematic review [19] in Asia identified some healthcare factors that were barriers to early breast cancer diagnosis and treatment but could not dissect the specific healthcare barriers in detail therefore creating a knowledge gap. Building on this knowledge gap of the above study [19] and also due to the weak healthcare systems in most LMICs, it is crucial to assess factors that are beyond the control of the individual such as the health system factors contributing to delay in the diagnosis and treatment of breast cancer. Providing a detailed description of the health system factors that influence the timely diagnosis of breast cancer using the World Health Organization (WHO) Health Systems Framework would provide clinicians and policymakers with key specific areas to strengthen and ensure early diagnosis and treatment of breast cancer. To the best of our knowledge, no study has adopted the WHO Health Systems Framework’s six building blocks [20] to itemize the key health system factors affecting the timely diagnosis and treatment of breast cancer among Asian women in developing countries. Classifying the findings under the WHO’s six building blocks (health service delivery, health workforce, health information system, access to essential medicines and technologies, health system financing, and leadership and governance), would provide a broader view of which health system factors requires agent intervention to improve timely diagnosis and treatment. Therefore, this systematic review comprehensively synthesized evidence on health system-related factors affecting the timely breast cancer diagnosis and treatment of Asian women.

## Methods

This review was systematically conducted in accordance with the Joanna Briggs Institute (JBI) methodology for systematic reviews and reported following the Preferred Reporting Items for Systematic Reviews and Meta-Analyses (PRISMA) guidelines [21]. The protocol for this systematic review was not registered.

### Data source and search strategy

We conducted a comprehensive search for relevant studies in the following electronic databases: PubMed, CINAHL, EMBASE, Web of Science, and PsycINFO. We adopted the three-step strategy proposed by JBI for all types of reviews [22]. Firstly, an initial limited search was undertaken in PubMed and CINAHL. To identify the keywords and index terms, we analyzed the text words within the title and abstract and index words that were used to describe the articles. Secondly, using the identified keywords (breast neoplasm, breast cancer, late diagnosis, delayed diagnosis, early diagnosis, and treatment) and index terms, a general search across the databases was performed. Thereafter, an individual search strategy for each database considering the differences in Thesaurus terminology and indexing was developed. Thirdly, hand search through Google and tracing of references of all articles included were searched for additional studies. A detailed search strategy for the various databases is attached in Additional file 1. The search was limited to articles published from 01-2012 to 01-04-2022. The year limit was chosen to gather current evidence on health system factors affecting the timely diagnosis and treatment of breast cancer. This systematic review elements formatting structure, PICOTS (Population-Intervention-Comparator-Outcome-Timing-Setting) for the development of eligibility criteria for the review (Table 1).

**Table 1:** Population-Intervention-Comparator-Outcome-Timing-Setting (PICOTS)

Study Component	Criteria
**Population**	Women in Asia medically diagnosed with breast cancer (of any age group)
**Intervention**	Breast cancer diagnosis and treatment
**Comparator**	Where applicable
**Outcome**	Health system factors influencing early detection, diagnosis, and treatment
**Timing**	01-01-2012 to 01-04-2022
**Setting**	Health facilities or hospitals in Asia

### Inclusion and exclusion criteria

The inclusion criteria involved studies that (1) reported primary research findings among women with breast cancer in Asia, (2) addressed health system factors influencing early detection, diagnosis, and treatment of breast cancer, and (3) were published in the English language from 2012 to 2022. Exclusion criteria included studies (1) without abstracts or full text and (2) undertaken outside of the Asian continent.

### Study selection

**Fig. 1:**
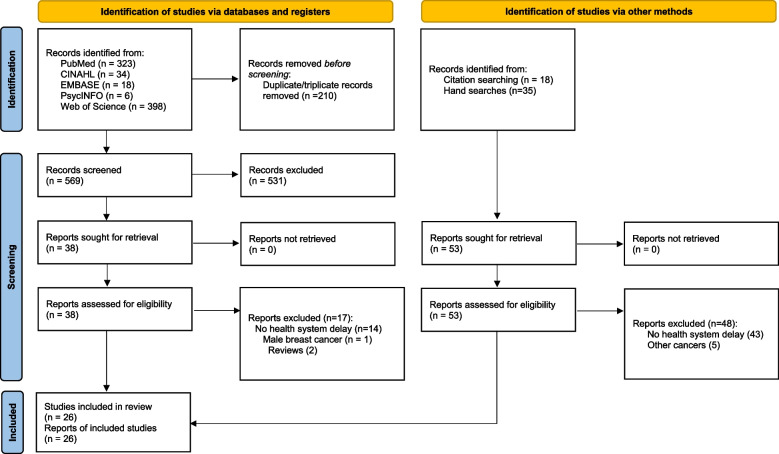
PRISMA flow diagram

Following the search of the electronic databases, all citations of the identified records were collated and uploaded into the EndNote X9 reference manager for removal of duplicated files and storage. The titles and abstracts of the studies were exported into a word file for screening. Two independent review authors (AA & RAA) screened the study titles and abstracts for relevance. The two reviewers then reconciled the outcome of the screening. Potential articles that seemed relevant for the review were retrieved in full. Thereafter, full-text articles were screened by two independent reviewer authors (AA & RAA) against the inclusion criteria. Full-text articles that did not meet the inclusion criteria were excluded and reasons for exclusion were justified. Any disagreements that occurred between the two review authors were resolved through mutual discussion, and where no consensus was reached, a third reviewer (RAK) was involved. The review articles selection process is detailed in the PRISMA 2020 flow diagram (Fig. 1).

### Data extraction

Two review authors (RAA and SDD) independently extracted data such as study characteristics (first authors, publication year, country, study aim and design, age group, participants, and sample size), and health system factors (barriers) [Table 2, Table 3]. The review adopted the WHO Health Systems Framework’s six building blocks [20] to extract and classify the health system factors. They include (a) health service delivery, (b) health workforce, (c) health information system, (d) access to essential medicines and technologies, (e) health system financing, and (f) leadership and governance. We then adopted the six building blocks because they are key contributing factors to health system strengthening in many ways. The key building blocks of WHO provide the basis for the overall policy and regulation of all the other health system factors. Crucial components of a successful health system include financing and the health workforce. Also service delivery and medical products and technologies reflect the immediate outputs of the health system, specifically the availability and distribution of care. The leadership and government component of the WHO building block was not utilized in this study as we did not identify related factors. Discrepancies in the study selection process and extraction were resolved through mutual discussion, with two other authors (MAA & RAK).

**Table 2:** Characteristics of studies

Characteristics		Frequency	Reference
**Total population**		**4457**	
**Publication year**			
2012–2016		7	[23–29]
2017–2022		19	[30–48]
**Category of Country**			
South Asia	Pakistan	10	[24, 30, 33, 34, 37, 38, 41, 44–46]
	Iran	2	[29, 39]
	India	5	[23, 31, 42, 43, 47]
	Bangladesh	1	[48]
Southeast Asia	Malaysia	2	[27, 28]
	Singapore & Malaysia	1	[25]
	Thailand	1	[26]
	Vietnam	2	[36, 40]
	Indonesia	1	[32]
Western Asia	Iraq	1	[35]
**Type of Study**			
Cross-sectional		15	[23, 24, 26, 31, 33–37, 41–46]
Qualitative		9	[25, 27–30, 32, 38–40, 49, 50]
Mixed method		2	[47, 48]

**Table 3:** Characteristics and results of included studies

First Author (year)	Country	Study aim	Study design	Sample	Key findings	WHOs framework
Agha et al., (2021) [30]	Pakistan	To explore and analyze knowledge, geographical and financial barriers, and how these barriers intersect and complicate BC patients’ lives.	Exploratory qualitative study	42 breast cancer survivors	Inadequate health care services. Cost of treatment. Poor quality healthcare. No diagnostic services are available (X-rays, scans). Long-distance.	Service delivery Health system financing Essential medicines and technology
D’almeida et al., (2021) [31]	India	To identify the barriers among Indian women diagnosed with breast cancer in an advanced stage.	Cross-sectional study	202 breast cancer patients	The non-availability of health resources nearby. Lack of specialty hospital. Nonavailability of female doctors	Health workforce
Dewi et al., (2021) [32]	Indonesia	To explore the psychosocial determinants of early presentation among female breast cancer survivors.	Exploratory qualitative study	23 breast cancer survivors	Previous health-related experience. and risk perceptions. Health care provider factors. Cost of treatment. Absence of a doctor.	Service delivery Health system financing Health workforce
Hussain, et al., (2021) [33]	Pakistan	To quantify total delay, provider delay, and patient delay, along with the factors contributing to each type of delay in breast cancer management	Cross-sectional study	334 breast cancer patients	Misdiagnosis. Appointments canceled before diagnosis was made.	Health workforce
Majeed et al., (2021) [34]	Pakistan	This study explores factors causing diagnostic and treatment delays among breast cancer patients.	Cross-sectional study	372 breast cancer patients	Referral delay and oncologist delay.	Health workforce
Mjali et al., (2021) [35]	Iraq	To describe presenting symptoms, risk factors, and medical care delays among breast cancer patients in Iraq.	Retrospective descriptive study	101 women with breast cancer	Non-availability of doctors.	Health workforce
Nguyen et al., (2021) [36]	North Vietnam	To understand the reasons and barriers to patients’ delay in seeking medical care are critical to mitigating the problem.	Cross-sectional study	462 breast cancer women	Cost of treatment. Long-distance to hospital.	Service delivery Health system financing
Rahool et al., (2021) [37]	Pakistan	The study aimed to evaluate the factors responsible for the delay in diagnosis of BC in Sindh, Pakistan	Cross-sectional study	197 breast cancer patients	Inaccessibility to healthcare. Unavailability of a female doctor. Appointment delays.	Service delivery Health workforce
Saeed et al., (2021) [38]	Pakistan	To identify and explore the barriers that hinder women from seeking timely screening and treatment.	Descriptive exploratory qualitative study	54 breast cancer women	Limited cancer screening centers. Lack of health professionals. Hospitals do not have cancer. Screening facilities. Lack of infrastructure. Poor attitude of paramedical staff and hospital administration. Cost of treatment. Lack of health insurance. Lack of female doctors.	Service delivery Health workforce Health system financing
Hossaini et al., (2020) [39]	Iran	The study aimed at identifying the barriers to the early detection of breast cancer in Iranian women.	Qualitative study	20 participants (11 breast cancer patients and 9 health professionals)	Low quality of diagnostic and medical services. Physician’s misdiagnosis of Mammography. Inadequate insurance coverage. The low number of female specialists, Lack of high-quality diagnostic Equipment and techniques, High treatment costs, Lack of easy access to services	Health workforce, Service delivery, access Essential medicines and technology, Health system financing
Jenkins et al., (2020) [40]	Vietnam	To understand, describe and analyze the experiences of women with breast cancer in Vietnam when accessing and using breast cancer services	Descriptive qualitative research design	13 women with breast cancer	Cost of treatment. Misdiagnosis. Poor communication Very complicated surgical biopsies. Being conducted without anesthesia. Overcrowding in the hospitals. Low physician/patient ratio.	Health system financing Health workforce Service delivery
Shamsi, et al., (2020) [41]	Pakistan	To evaluate the frequency and length of delays in seeking medical consultation and to assess the factors associated with them.	Cross-sectional study	499 breast cancer patients	No action was taken by healthcare providers. Wrongly reassured about the lump without mammography or biopsy.	Health workforce
Shreyamsa et al., (2020) [42]	India	To identify patient-perceived barriers to BC management.	Cross-sectional study	435 breast cancer patients	Misdiagnosis first consultation. Delays in referral by first contact practitioner. Distance traveled to avail expert services. Lack of information about available health facilities. Waiting periods at hospitals. Absence of female doctors.	Service delivery Health workforce Health information systems
Somanna et al., (2020) [43]	India	To delineate the time interval between self-detection of breast cancer symptoms and seeking care and to find the main reasons for the delay in seeking care	Cross-sectional study design	181 breast cancer patients	Distance of tertiary care center. Multiple medical practitioners who did not suspect cancer.	Service delivery Health workforce
Baig et al., (2019) [44]	Pakistan	To identify the factors responsible for the delayed presentation of patients with breast carcinoma	Cross-sectional study	89 breast cancer patients	Non-availability of health care facilities.	Service delivery
Gulzar et al., (2019) [45]	Pakistan	To identify the reasons for delayed presentation and their association with various sociodemographic variables.	Cross-sectional study	125 breast cancer patients	The hospital was too far away.	Service delivery
Hameed Khaliq et al., (2019) [46]	Pakistan	To determine association of various socio-demographic and clinical indicators on the length of breast cancer patients’ time before reaching diagnostic facilities.	Cross-sectional study	200 patients with breast cancer	Misguided by the doctor.	Health workforce
Kumar et al., (2019) [47]	India	To estimate the overall delay in diagnosis and treatment of breast cancer and the associated factors, describe the pathway of care, and explore the reasons for delay from a patient’s and providers’ perspective	Mixed method study	269 breast cancer patients	Poor accessibility. Delay in diagnosis of breast cancer. The long waiting list for surgery.	Service delivery Health workforce
Steiness et al., (2018) [48]	Bangladesh	To identify barriers to care for women with breast cancer symptoms in rural Bangladesh	Mixed method study	43 breast cancer patients and 20 men	We do not have enough expert doctors. Lack of the necessary machines. Inadequate technicians. Distance to a facility. Bad interpersonal experience with a doctor.	Service delivery Access to essential medicines and technologies Health workforce
Gangane et al., (2016) [23]	India	To examine the extent of diagnosis delay among breast cancer patients and to identify the underlying risk factors associated with the delay.	Cross-sectional study	212 breast cancer patients	High hospital costs.	Health system financing
Khan et al., (2015) [24]	Pakistan	To determine causes of delayed presentation and to determine the association of delayed presentation with age, family history, marital, menopausal, education, and socioeconomic status.	Cross-sectional study	315 breast cancer patients	No access to female doctors. Cost of treatment.	Service delivery Health workforce Health system financing
Lim et al., (2015) [25]	Singapore and Malaysia	To explore and compare barriers to the early presentation of self-discovered breast cancer	Qualitative study	67 women with breast cancer	Poor quality of care and services. Misdiagnosis. Expensive treatment cost. [Findings from the Malaysian context are extracted for this review]	Service delivery Health workforce Health system financing
Poum et al., (2014) [26]	Thailand	To identify factors associated with delayed first consultation for breast symptoms, delayed diagnosis after the first consultation, and advanced pathologic stage at presentation in 180 women with breast cancer in Thailand.	Cross-sectional study	180 breast cancer women	Increased number of consultations with a surgeon before diagnosis. Greater time to referral.	Health workforce
Taib et al., (2014) [27]	Malaysia	To explore the reasons why women present with breast cancer at an advanced stage	Qualitative study	19 breast cancer patients	Poor recognition of breast cancer signs and symptoms. Misinformed about its malignancy and contradicting information. Recommendations from different health care providers. Ineffective communication by health care providers. Not referred to a diagnostic facility.	Health workforce Health information
Norsa’adah et al., (2012) [28]	Malaysia	To explore reasons for the delay in seeking help among patients with breast cancer	Qualitative study	12 breast cancer women	Weak health care systems. Misdiagnosis. No appointment was given.	Service delivery
Rastad et al., (2012) [29]	Iran	To gain insight into the causes of delay in seeking treatment in patients with breast cancer.	Qualitative study	10 women with breast cancer	Physicians giving the wrong diagnosis.	Health workforce

### Data synthesis

The review underpinned a narrative synthesis approach without meta-analysis [51]. A meta-analysis was not possible due to the heterogeneity of the study designs and the variability of the outcome measures. Thus, the narrative synthesis approach was deemed useful as the aim of this review was to primarily identify the health system factors that affected the early detection, diagnosis, and treatment of breast cancer using the six building blocks of the WHO Health Systems Framework. The extracted information from the studies was read and reread to identify the health system factors influencing timely breast cancer diagnosis and treatment. We categorized each health system factor in line with the six building blocks. Each building block then form a major category. We then provided a narrative approach to present the findings, including tables and figures to aid in data presentation, where appropriate.

### Assessment of methodological quality

The mixed Methods Appraisal Tool (MMAT) [52] was utilized to evaluate and appraise the qualitative, quantitative, and mixed methods research designs. The MMAT assesses the appropriateness of the study aim and design, methodology, participant recruitment, data collection, data analysis, presentation of findings, authors’ discussions, and conclusions. Two review authors (RAA & VNY) independently reviewed and appraised the articles (see Additional file 2). Discrepancies regarding the quality assessment of the articles included were discussed among all the authors to resolve disagreements.

## Results

An initial search through the electronic databases yielded 779 studies, of which 210 duplicates/triplicates articles were removed. A total of 569 non-duplicate studies were screened by title and abstract and 531 studies were excluded using the exclusion criteria. Of the remaining 38 studies, 21 articles met the inclusion criteria. An additional five articles were included through reference tracing and hand searches. Finally, a total of 26 studies were included in this review (Fig. 1).

### Characteristics of the included studies

Of the 26 [23–48] studies included in this review, 15 used quantitative [23, 24, 26, 31, 33–37, 41–46], nine used qualitative [25, 27–30, 32, 38–40], and two used mixed-methods design [47, 48]. The cumulative sample size of breast cancer patients was 4457. The sample sizes of the studies ranged from 10 to 499 for the quantitative, qualitative, and mixed studies. The studies were conducted across 10 countries in Asia: 18 studies are from South Asia [23, 24, 29–31, 33, 34, 37–39, 41–48], seven studies are from Southeast Asia [25–28, 32, 36, 40], and a study from Western Asia [35] (see Table 2).

### Quality assessments outcome

Quality assessment of the studies reviewed showed that most (fifteen) of the studies were well-designed to reduce bias. However, most (eleven) of the included quantitative descriptive studies did not use validated tools as they did not indicate the validity and reliability of the measurements used. The risks of non-response bias could not be assessed as a few studies (four) did not report the non-response rates. The two mixed-method studies did not provide an adequate rationale for using a mixed-methods design to address the research question. As MMAT discourages the calculation of the overall score from the ratings of each criterion, reviewers did not assign any scores for appraisal [52] (see Additional file 2).

### Health system barriers to timely breast cancer diagnosis and treatment

**Fig. 2:**
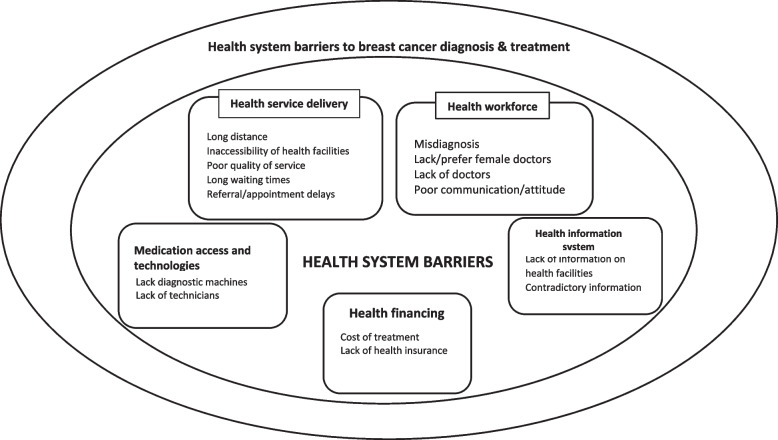
Key health system barriers to breast cancer diagnosis and treatment among Asian women

We categorized the barriers to timely breast cancer diagnosis and treatment into five sections which include (1) delivery of health services (2) health workforce (3) financing for health (4) health information system (5) essential medicines and technology (see Table 4, Fig. 2).

**Table 4:** Health system barriers classified according to WHO building blocks

WHO building blocks	Health system barriers	References
Health Service delivery	Long-distance	[30, 36, 39, 42, 43, 45, 48]
	Non-availability of healthcare facilities	[30, 31, 37, 44]
	Referral delay	[27, 34, 42]
	Long waiting times	[42, 47, 50]
	Poor quality of care and services	[25, 28, 30, 38]
	Appointment delays	[28, 33, 37, 38]
	Procedures at hospitals are very complicated	[40]
	Limited cancer screening centers	[38]
	Conducting biopsies without anesthesia	[40]
	Overcrowding in the hospitals	[40]
	Weak healthcare systems	[28]
Health workforce	Misdiagnosis	[25, 28, 29, 33, 39, 40, 42]
	Lack/prefer female doctors	[24, 37–39, 42, 50]
	Lack of doctors/expert doctors	[32, 35, 38, 40, 48]
	Poor communication	[27, 40]
	Poor attitude of paramedical staff and hospital administration	[38]
	No action was taken by the providers	[41]
	Multiple medical practitioners who did not suspect cancer	[43]
	Bad interpersonal experience with a doctor	[48]
	Misguided by the doctor	[46]
	Poor recognition of breast cancer signs and symptoms	[27]
	Healthcare provider factors	[32]
Financing for health	Cost of treatment	[23–25, 30, 32, 36, 38–40, 49, 50]
	Lack of health insurance	[38, 39]
Information system	Lack of information about available health facilities.	[42]
	Provision of contradicting information	[41]
Essential medicine and technology	No diagnostic services available (X-rays, scans)	[30]
	Lack of infrastructure	[38]
	Lack of the necessary machines	[48]
	Inadequate technicians	[48]
	Low quality of diagnostic and medical services	[39]

### Delivery of health services

Nineteen studies addressed health service delivery. The review identified that low care quality and service delivery within health facilities contributed to significant delays in Asian women seeking breast cancer treatments [25, 30, 39]. A study among Iranian women reported:

*“The low quality of medical services decreases timely visits. … some women, who visited a physician on time, underwent mastectomy, or passed away, or had a low survival chance due to errors in medical practices. This reduces the patients’ trust in the medical sector”* [39]*.*

Complicated procedures such as surgical biopsies were conducted without anesthesia, thus inducing pain [40] as were the main factors influencing delays in seeking healthcare. Other factors also include overcrowding [40] and long waiting times [38, 42, 50] at the consultation facilities. A long waiting list for surgery [47] was a factor contributing to delays among women with breast cancer in seeking prompt treatment. We identified that women who were not given appointments [28] and those who experienced appointment delays [33, 37, 38] in health facilities were likely to seek late or even quit treatment. For example, a woman narrated:

“… *I was forced to wait for more than 3 hours even though I had an appointment. Sometimes the behavior of staff also discouraged me, and I thought that I should quit treatment”* [38]*.*

Long-distance and a lack of access to hospitals [30, 36, 39, 43, 45, 48], limited cancer screening centers [38], and hospitals not having screening facilities were predominant factors influencing delays in seeking diagnosis and treatment. Inaccessibility [37] and the non-availability of healthcare facilities [31] were barriers to the early diagnosis and treatment of breast cancer among women. Referral delays by medical practitioners [26, 27, 34, 42] were also identified as barriers to early diagnosis and treatment.

### Health workforce

Eighteen studies identified health workforce. Among the health workforce factors, the unavailability of doctors [32, 35], especially oncologists and cancer specialists [38, 48] served as a barrier to early diagnosis and treatment of breast cancer. Several studies reported the lack and preference for female doctors over male doctors [24, 31, 37–39, 42]. A study in Pakistan indicated that women were reluctant to visit male doctors for breast cancer treatment as they did not want to expose their bodies to male doctors [38]. A woman narrated:

*“… it was very embarrassing for me to discuss my breasts with him. It was worse when he asked me to show the tumor. As a Muslim female, I could not imagine that I can ever show my breasts to an unknown male …*” [38].

A low physician-patient ratio was also identified as a barrier to early diagnosis and treatment of breast cancer [40]. Delay in diagnosis [47] and misdiagnosis [25, 28, 29, 33, 39, 40, 42] resulted in one of the most common health workforce factors that served as a barrier to the early detection of breast cancer. For example, a woman narrated:

“*The doctors did not diagnose a tumor. They said everything was okay”* [40].

Healthcare providers not being able to recognize breast cancer signs and symptoms [27], and not suspecting breast cancer by multiple medical practitioners [43] served as an added barrier to early diagnosis and treatment. Poor communication and misinformation by professionals [27, 40], poor attitudes of paramedical staff, hospital administration [38], and a lack of interpersonal experience with health professionals [48] served as health workforce factors influencing delayed treatment. A participant narrated:

*“Initially, I faced a terrible experience when I visited a renowned hospital … They made me feel like a burden and I realized that old people are not needed by society … their (physicians’) attitude made my sickness worst.”* [38].

### Financing for health

Eleven studies identified health financing. Two major factors were identified as health financing-related factors: (1) the high cost of hospital treatments and investigations and (2) a lack of health insurance scheme support. High costs of hospital treatment and investigations were reported as an important factor influencing women’s access to breast cancer treatment in 11 studies [23–25, 30, 32, 36, 38–40]. Another health financing-related factor was the lack of insurance [38], and inadequate insurance coverage [39] serving as a barrier to early diagnosis and treatment of breast cancer.

### Essential medicines and technology

Three studies identified essential medicines and technologies. This review identified a lack of the necessary breast cancer diagnostic equipment [48], while some studies indicated the non-availability of diagnostic services [30] and low quality of diagnostic and medical services [39].

### Health information system

Among all the included studies, only two studies from developing countries addressed information systems [41, 42]. One of the studies revealed that the lack of information about available health facilities was a perceived barrier among Indian women in a poor resource setting [42]. Women who received contradicting information about breast cancer from different health professionals were most likely to present late for breast cancer treatment [41].

## Discussion

This mixed-methods systematic review provides evidence of literature on health system-related barriers influencing timely breast cancer diagnosis and treatment of Asian women in LMICs. The findings of this study can serve as directions for relevant stakeholders and clinicians to design preemptive models against elements that cause delay and promote timely diagnosis and treatment in Asia.

An experience of low-quality health care and service delivery could deter patients from seeking early medical care in health facilities. Similarly, several studies outside the Asian region have also reported misdiagnosis, failure to suspect cancer at the initial consultation [53], medical errors, and false-negative interpretations of mammography [54–56] as a result of provider delay in breast cancer diagnosis, and treatment. In developing countries, where highly specialized human resources are scarce, and lack of diagnostic equipment might be attributed [57] to inaccurate medical diagnoses. Also, our finding resonate with a recent systematic review conducted in sub-Saharan Africa (SSA) that attributed misdiagnosis to poor knowledge and inadequate training of health care professionals on breast cancer [57]. The long waiting times and overcrowding in health facilities lead to dissatisfaction which in turn, leads to delays in the diagnosis and treatment of breast cancer among women. Overcrowding, long waiting times in health facilities, lack of cancer care facilities and scarcity of human resources were primary concerns of developing Asian countries as observed in this study. Consistent with our findings, a study reported geographical inaccessibility of different breast cancer treatment services as a key barrier to care in SSA [57]. Similarly, in LMICs, lack of accessibility to primary care and inability to obtain appointments with health care professionals were barriers to breast cancer care [58]. This resonated with access to diagnostics and geographical limitations to timely diagnosis and treatment. The findings suggest that efforts are required by policymakers to ensure availability of breast cancer screening and treatment centers that are easily accessible to breast cancer patients amid global surge in the number of breast cancer incidences.

Provision of culturally sensitive care by recognizing unique cultural, religious, and social beliefs and practices is of paramount importance. Globally, women’s cultural perceptions and attitudes towards breast cancer should be examined to optimize timely breast cancer diagnosis and treatment. A recent study conducted in New York city identified structural barriers (language, insurance status) and socio-cultural barriers (lack of knowledge on preventive cancer, gender roles, stigma, sex of doctors, and feelings of fatalism) as key barriers to breast and cervical cancer screening among Muslim women [59]. Similarly, a study conducted among Chinese women in the United Kingdom elicited social-cultural influences on the perceptions of breast cancer. Cancer is perceived as a taboo topic among Chinese women, and it is not recommended for discussion with others. Similar to the beliefs, Muslim women, Chinese and Filipino women associated cancer with an event that is predetermined by God, where external forces of humans have minimal power to influence [60, 61]. Prior to providing treatment care plans, it is important for healthcare professionals to critically examine the cultural values of Asian women in relation to breast cancer diagnosis and treatment [62]. Further research is warranted to illuminate knowledge on breast cancer and screening among ethnically diverse Southeast Asian women, as knowledge predicts greater intent for cancer screening. The development of culturally competent services involving language and culture-specific educational interventions would facilitate more women to proactively seek breast cancer screening, diagnosis, and treatment.

The cost of breast cancer treatment is considerable and persistently high beyond the affordability of most vulnerable populations. It even goes beyond the period of acute treatment to the advanced stages leading to medical debt deterring most patients from seeking care [63, 64]. Health financing-related factors were identified as barriers influencing early diagnosis and treatment of breast cancer. The high cost of breast cancer treatment and investigations were identified as key barriers to the early diagnosis and treatment of breast cancer. Our finding is consistent with recent systematic reviews conducted in Africa [57, 65]. The high cost of breast cancer diagnosis and treatment is observed in most countries worldwide, including developed and developing countries [66]. In some parts of Asia, the average out-of-pocket payment (OOP) for breast cancer diagnosis and treatment is 61.8 million VND ($2667) in Vietnam [67], while in China, the curative care expenditure for breast cancer was also similar [68]. The studies indicated that the advancement of the stage of cancer at diagnosis is associated with an increased cost. Therefore, in order to improve early diagnosis and treatment and to reduce the burden of breast cancer, interventions targeting subsidizing the high cost of treatment and policies aimed at early detection to reduce both health and economic impacts of breast cancer are imperative.

Holding health insurance grants individuals access to affordable health care. Some studies reported that uninsured patients were most likely to delay or forgo cancer care and prescribed medication due to its high costs [69–71]. Although health insurance is a key determining factor of access to health care, having insurance does not always translate to affordable care [72]. In most countries, health insurance does not cover all costs of cancer treatment and therefore forces women to pay out of pocket for services rendered in return, hence discouraging women from seeking treatment. The findings of this review are congruent with a review conducted in the Middle East and North Africa (MENA) region [73]. The study conducted in the MENA region found the largest increased odds for a mammogram were from women having insurance [73]. This study found that delayed diagnosis and treatment of breast cancer was more associated uninsured women. This study recommends expansion of the coverage of health insurance among women in LMICs to foster early diagnosis and treatment of breast cancer.

Cancer care challenge is a significant problem in developing countries. As a result of logistical and economic issues, where mammographic screening is limited, an early diagnosis of breast cancer in most developing countries is questionable [74]. Comparatively, breast cancer prognosis is better for women in developed countries than in developing countries. For instance, 70% of women in the United States undergo mammogram scans [75]. However, in the context of developing countries with budget limitations, breast cancer is often diagnosed with the presence of palpable mass during self-breast examination [76]. Studies have found that Asians diagnosed with breast cancer from low and middle-income countries were younger and more susceptible to dying from advanced stages of a cancer diagnosis. Sufficient funding is therefore required to establish cancer care systems offering breast cancer diagnostics, treatment, and palliative care services in LMICs of Asia [77].

Interestingly, this review did not identify related factors for leadership and governance domain of the WHO six building blocks. The leadership and governance systems, is a cross-cutting component, such that it provide the basis for the overall policy and regulation of all the other health system blocks [20]. The lack of this important component calls for attention to this key and important sector of the health care system. Most LMICs lack policies or cancer control strategies which further influence the burden of the disease in these countries. We recommend that future studies should assess the leadership and governance component of the six building blocks to enable health system strengthening and for policy implementations by policymaker and relevant stakeholders.

### Implications for health systems policies and research

This study showed how health system barriers influence the timely diagnosis and treatment of breast cancer among women in Asian countries. The outcome of this study has several implications for health system policies and research. There is a need to establish deliberate barrier-free policies that will prioritize the timely diagnosis of women with breast cancer in Asia. The policies should further encourage the promotion of user-friendly cancer treatments for women who have already been diagnosed with breast cancer in Asia. We recommend that policies should target increasing the number of skilled and specialised healthcare professionals (doctors & nurses etc) and breast cancer screening centers to promote timely diagnosis and treatment Asia. Health care systems in LMICs in Asia should prioritise the needs of women preferences during seeking health care especially increasing the availability of female specialist doctors that provide breast cancer diagnosis and treatment services. In the same vein, health system research should carefully prioritize the socio-cultural differences in promoting early breast cancer diagnosis and treatment among women in Asian countries.

### Strengths and limitations of this study

This study has some strengths and limitations worth noting. One of the key strengths of this study is the use of the WHO health systems’ six building blocks to categorize the health system barriers to timely breast cancer diagnosis and treatment. Also, a comprehensive search in several databases provided several relevant studies for this review. However, the results may not be transferable to all Asian countries as Asia comprises both LMICs and developed nations. Additionally, as studies published in any language other than English were excluded from this review, pertinent information potentially published in other languages could have been missed. Despite the limitation, this study highlights important findings of how Asian countries can work in partnerships to improve infrastructure and resources in LMICs of Asia.

## Conclusion

This systematic review presented a comprehensive overview of the underlying health system challenges confronting women with breast cancer in accessing treatments in health facilities within Asia. The key health system barriers identified are high cost and geographical accessibility to breast cancer treatment, misdiagnosis from health professionals, lack of doctors and preference for female doctors, referral delays, and long waiting times at health facilities. These findings provide an opportunity for the strengthening of health systems and the implementation of suitable interventions to ensure these barriers are adequately eliminated to promote timely diagnosis and treatment of breast cancer among women in Asia.

## Supplementary Information

**Additional file 1.** Search strategies.

**Additional file 2 **Mixed Methods Appraisal Tool (MMAT) in selected studies (*N*=26).

## Data Availability

All data generated or analysed during this study are included in this published article [and its supplementary information files].

## Abbreviations

CNY: Chinese yuan renminbi
JBI: Joanna Briggs Institute
LMICs: Low-and Middle-Income Countries
MENA: Middle East and North Africa
MMAT: Mixed Methods Appraisal Tool
OOP: Out-of-Pocket Payment
PICOTS: Population-Intervention-Comparator-Outcome-Timing-Setting
PRISMA: Preferred Reporting Items for Systematic Reviews and Meta-Analyses
SSA: sub-Saharan Africa
VND: Vietnamese Dong
WHO: World Health Organization
