# Supplementary material for: Health system barriers influencing timely breast cancer diagnosis and treatment among women in low and middle-income Asian countries: evidence from a mixed-methods systematic review
Source: BMC Health Serv Res. 2022 Dec 31;22:1601. doi: 10.1186/s12913-022-08927-x (PMC9805268; doi:10.1186/s12913-022-08927-x)
Supplement: Supplementary file 1 — Additional file 1. Search strategies. [file 12913_2022_8927_MOESM1_ESM.docx]

**Additional file 1: Search strategies**

|  |  |  |  |
| --- | --- | --- | --- |
| **Database** |  | **Search strategies** | **Results** |
| **PubMed** | **#1** | "Breast Neoplasms"[MeSH Terms] OR "Breast Neoplasms" OR "Breast cancer" OR "Breast Neoplasia" OR "Breast Tumor" OR “Breast carcinoma” | 198,201 |
|  | **#2** | “Breast cancer survivor*” OR “Breast cancer patient*” | 30,586 |
|  | **#3** | “Delayed treatment” OR “early diagnosis” OR “timely diagnosis” OR “delayed diagnosis” OR “late Presentation” OR “early presentation” OR “early detection” | 140,580 |
|  | **#4** | Factors OR Determinants OR Barriers OR Challenges | 6,472,982 |
|  | **#5** | Asia OR China OR India OR Indonesia OR Pakistan OR Bangladesh OR Japan OR Philippines OR Vietnam OR Turkey OR Iran OR Thailand OR Myanmar OR South Korea OR Iraq OR Afghanistan OR Saudi Arabia OR Uzbekistan OR Malaysia OR Yemen OR Nepal OR North Korea OR Sri Lanka OR Kazakhstan OR Syria OR Cambodia OR Jordan OR Azerbaijan OR United Arab Emirates OR Tajikistan OR Israel OR Laos OR Lebanon OR Kyrgyzstan OR Turkmenistan OR Singapore OR Oman OR Palestine OR Kuwait OR Georgia OR Mongolia OR Armenia OR Qatar OR Bahrain OR Cyprus OR Bhutan OR Maldives OR Brunei | 4,017,856 |
|  | **#6** | **#1AND#2AND#3AND#4AND#5** | **323** |
|  |  |  |  |
|  | **#1** | “Breast Neoplasms" OR "Breast Neoplasms" OR "Breast cancer" OR "Breast Neoplasia" OR "Breast Tumor" OR “Breast carcinoma” | 384,730 |
|  |  | ('breast neoplasms'/exp OR 'breast neoplasms' OR 'breast cancer'/exp OR 'breast cancer' OR 'breast neoplasia'/exp OR 'breast neoplasia' OR 'breast tumor'/exp OR 'breast tumor' OR 'breast carcinoma'/exp OR 'breast carcinoma') AND [01-01-2012]/sd NOT [01-04-2022]/sd |  |
|  | **#2** | “Breast cancer survivor*” OR “Breast cancer patient*” | 57,294 |
|  |  | ('breast cancer survivor*' OR 'breast cancer patient*') AND [01-01-2012]/sd NOT [01-04-2022]/sd |  |
|  | **#3** | “Delayed treatment” OR “early diagnosis” OR “timely diagnosis” OR “delayed diagnosis” OR “late Presentation” OR “early presentation” OR “early detection” | 194,098 |
|  |  | ('delayed treatment' OR 'early diagnosis'/exp OR 'early diagnosis' OR 'timely diagnosis' OR 'delayed diagnosis'/exp OR 'delayed diagnosis' OR 'late presentation' OR 'early presentation' OR 'early detection') AND [01-01-2012]/sd NOT [01-04-2022]/sd | 194,098 |
|  | **#4** | Factors OR Determinants OR Barriers OR Challenges |  |
|  |  | (factors OR 'determinants'/exp OR determinants OR 'barriers'/exp OR barriers OR challenges) AND [01-01-2012]/sd NOT [01-04-2022]/sd | 2,343,277 |
|  | **#5** | (((((('asia'/exp OR asia OR 'china'/exp OR china OR 'india'/exp OR india OR 'indonesia'/exp OR indonesia OR 'pakistan'/exp OR pakistan OR 'bangladesh'/exp OR bangladesh OR 'japan'/exp OR japan OR 'philippines'/exp OR philippines OR 'vietnam'/exp OR vietnam OR 'turkey'/exp OR turkey OR 'iran'/exp OR iran OR 'thailand'/exp OR thailand OR 'myanmar'/exp OR myanmar OR south) AND ('korea'/exp OR korea) OR 'iraq'/exp OR iraq OR 'afghanistan'/exp OR afghanistan OR 'saudi'/exp OR saudi) AND ('arabia'/exp OR arabia) OR 'uzbekistan'/exp OR uzbekistan OR 'malaysia'/exp OR malaysia OR 'yemen'/exp OR yemen OR 'nepal'/exp OR nepal OR north) AND ('korea'/exp OR korea) OR sri) AND lanka OR 'kazakhstan'/exp OR kazakhstan OR 'syria'/exp OR syria OR 'cambodia'/exp OR cambodia OR 'jordan'/exp OR jordan OR 'azerbaijan'/exp OR azerbaijan OR united) AND ('arab'/exp OR arab) AND emirates OR 'tajikistan'/exp OR tajikistan OR 'israel'/exp OR israel OR 'laos'/exp OR laos OR 'lebanon'/exp OR lebanon OR 'kyrgyzstan'/exp OR kyrgyzstan OR 'turkmenistan'/exp OR turkmenistan OR 'singapore'/exp OR singapore OR 'oman'/exp OR oman OR 'palestine'/exp OR palestine OR 'kuwait'/exp OR kuwait OR 'georgia'/exp OR georgia OR 'mongolia'/exp OR mongolia OR 'armenia'/exp OR armenia OR 'qatar'/exp OR qatar OR 'bahrain'/exp OR bahrain OR 'cyprus'/exp OR cyprus OR 'bhutan'/exp OR bhutan OR 'maldives'/exp OR maldives OR 'brunei'/exp OR brunei) AND [01-01-2012]/sd NOT [01-04-2022]/sd | 627,830 |
|  |  | **1AND#2AND#3AND#4AND#5** | **18** |
| **CINAHL** | **#1** | “Breast Neoplasms" OR "Breast Neoplasms" OR "Breast cancer" OR "Breast Neoplasia" OR "Breast Tumor" OR “Breast carcinoma” | **65,115** |
|  | **#2** | “Breast cancer survivor*” OR “Breast cancer patient*” | **11,210** |
|  | **#3** | “Delayed treatment” OR “early diagnosis” OR “timely diagnosis” OR “delayed diagnosis” OR “late Presentation” OR “early presentation” OR “early detection” | **43,592** |
|  | **#4** | Factors OR Determinants OR Barriers OR Challenges | **1,218,891** |
|  | **#5** | Asia OR China OR India OR Indonesia OR Pakistan OR Bangladesh OR Japan OR Philippines OR Vietnam OR Turkey OR Iran OR Thailand OR Myanmar OR South Korea OR Iraq OR Afghanistan OR Saudi Arabia OR Uzbekistan OR Malaysia OR Yemen OR Nepal OR North Korea OR Sri Lanka OR Kazakhstan OR Syria OR Cambodia OR Jordan OR Azerbaijan OR United Arab Emirates OR Tajikistan OR Israel OR Laos OR Lebanon OR Kyrgyzstan OR Turkmenistan OR Singapore OR Oman OR Palestine OR Kuwait OR Georgia OR Mongolia OR Armenia OR Qatar OR Bahrain OR Cyprus OR Bhutan OR Maldives OR Brunei | **277,558** |
|  | **#6** | **1AND#2AND#3AND#4AND#5** | **34** |
|  |  |  |  |
| **PSYCINFO** | **#1** | “Breast Neoplasms" OR "Breast Neoplasms" OR "Breast cancer" OR "Breast Neoplasia" OR "Breast Tumor" OR “Breast carcinoma” | **7,274** |
|  | **#2** | “Breast cancer survivor*” OR “Breast cancer patient*” | **2,413** |
|  | **#3** | “Delayed treatment” OR “early diagnosis” OR “timely diagnosis” OR “delayed diagnosis” OR “late Presentation” OR “early presentation” OR “early detection” | **9,380** |
|  | **#4** | Asia OR China OR India OR Indonesia OR Pakistan OR Bangladesh OR Japan OR Philippines OR Vietnam OR Turkey OR Iran OR Thailand OR Myanmar OR South Korea OR Iraq OR Afghanistan OR Saudi Arabia OR Uzbekistan OR Malaysia OR Yemen OR Nepal OR North Korea OR Sri Lanka OR Kazakhstan OR Syria OR Cambodia OR Jordan OR Azerbaijan OR United Arab Emirates OR Tajikistan OR Israel OR Laos OR Lebanon OR Kyrgyzstan OR Turkmenistan OR Singapore OR Oman OR Palestine OR Kuwait OR Georgia OR Mongolia OR Armenia OR Qatar OR Bahrain OR Cyprus OR Bhutan OR Maldives OR Brunei | **202,429** |
|  | **#5** | **#1 AND #2 AND # 3 AND #4** | **6** |
|  |  |  | **381** |
| **Web of science** | **#1** | “Breast Neoplasms" OR "Breast Neoplasms" OR "Breast cancer" OR "Breast Neoplasia" OR "Breast Tumor" OR “Breast carcinoma” | 321,183 |
|  | **#2** | “Breast cancer survivor*” OR “Breast cancer patient*” | 40,121 |
|  | **#3** | “Delayed treatment” OR “early diagnosis” OR “timely diagnosis” OR “delayed diagnosis” OR “late Presentation” OR “early presentation” OR “early detection” | 113,789 |
|  | **#4** | Asia OR China OR India OR Indonesia OR Pakistan OR Bangladesh OR Japan OR Philippines OR Vietnam OR Turkey OR Iran OR Thailand OR Myanmar OR South Korea OR Iraq OR Afghanistan OR Saudi Arabia OR Uzbekistan OR Malaysia OR Yemen OR Nepal OR North Korea | **10,525,844** |
|  | **#5** | Sri Lanka OR Kazakhstan OR Syria OR Cambodia OR Jordan OR Azerbaijan OR United Arab Emirates OR Tajikistan OR Israel OR Laos OR Lebanon OR Kyrgyzstan OR Turkmenistan OR Singapore OR Oman OR Palestine OR Kuwait OR Georgia OR Mongolia OR Armenia OR Qatar OR Bahrain OR Cyprus OR Bhutan OR Maldives OR Brunei | 1,235,641 |
|  |  | **#1AND#2AND#3AND#4AND#5** | **398** |
|  |  | **Total** | **779** |
