# Supplementary material for: Health system barriers influencing timely breast cancer diagnosis and treatment among women in low and middle-income Asian countries: evidence from a mixed-methods systematic review
Source: BMC Health Serv Res. 2022 Dec 31;22:1601. doi: 10.1186/s12913-022-08927-x (PMC9805268; doi:10.1186/s12913-022-08927-x)
Supplement: Supplementary file 2 — Additional file 2 Mixed Methods Appraisal Tool (MMAT) in selected studies (N=26). [file 12913_2022_8927_MOESM2_ESM.docx]

**Additional file 2**

*Mixed Methods Appraisal Tool (MMAT) in selected studies (N=26)*

|  | S1. | S2. | 4.1 | 4.2 | 4.3 | 4.4 | 4.5 |
| --- | --- | --- | --- | --- | --- | --- | --- |
| **Quantitative descriptive studies** | Are there clear research questions? | Do the collected data allow to address the research questions? | Is the sampling strategy relevant to address the research question? | Is the sample representative of the target population? | Are the measurements appropriate? | Is the risk of nonresponse bias low? | Is the statistical analysis appropriate to answer the research question? |
| D’almeida et al., (2021) | Yes | Yes | Yes | Yes | Can’t tell | Yes | Yes |
| Hussain, et al., (2021) | Yes | Yes | Can’t tell | Can’t tell | Can’t tell | Yes | Can’t tell |
| Majeed et al., (2021) | Yes | Yes | Yes | Yes | Can’t tell | No | Can’t tell |
| Mjali et al., (2021) | Yes | Yes | Can’t tell | Can’t tell | Can’t tell | No | Yes |
| Nguyen et al., (2021) | Yes | Yes | Yes | Yes | Yes | Yes | Yes |
| Rahool et al., (2021) | Yes | Yes | Yes | Yes | Can’t tell | Can’t tell | Yes |
|  |  |  |  |  |  |  |  |
| Shamsi, et al., (2020) | Yes | Yes | Yes | Yes | Yes | Yes | Yes |
| Shreyamsa et al., (2020) | Yes | Yes | Can’t tell | Yes | Can’t tell | Yes | Yes |
| Somanna et al., (2020) | Yes | Yes | Can’t tell | Yes | Can’t tell | Yes | Yes |
| Gulzar et al., (2019) | Yes | Yes | Can’t tell | Yes | Can’t tell | Yes | Yes |
| Hameed Khaliq et al., (2019) | Yes | Yes | Yes | Yes | Can’t tell | Yes | Yes |
| Baig et al., (2018) | Yes | Yes | Yes | Yes | Yes | Yes | Yes |
|  |  |  |  |  |  |  |  |
| Gangane et al., (2016) | Yes | Yes | Yes | Yes | Can’t tell | Yes | Yes |
| Khan et al., (2015) | Yes | Yes | Yes | Yes | Can’t tell | Can’t tell | Yes |
| Poum et al., (2014) | Yes | Yes | Yes | Yes | Yes | Yes | Yes |

**Quality assessment of Mixed method studies**

|  | S1. | S2. | 5.1 | 5.2 | 5.3 | 5.4 | 5.5 |
| --- | --- | --- | --- | --- | --- | --- | --- |
| **Mixed method studies** | Are there clear research questions? | Do the collected data allow to address the research questions? | Is there an adequate rationale for using a mixed-methods design to address the research question? | Are the different components of the study effectively integrated to answer the research question? | Are the outputs of the integration of qualitative and quantitative components adequately interpreted? | Are divergences and inconsistencies between quantitative and qualitative results adequately addressed? | Do the different components of the study adhere to the quality criteria of each tradition of the methods involved? |
| Kumar et al., (2019) | Yes | Yes | No | Yes | Yes | Yes | Yes |
| Steiness et al., (2018) | Yes | Yes | No | Yes | Yes | Yes | Yes |
|  | S1. | S2. | 1.1 | 1.2 | 1.3 | 1.4 | 1.5 |
| **Qualitative studies** | Are there clear research questions? | Do the collected data allow to address the research questions? | Is the qualitative approach appropriate to answer the research question? | Are the qualitative data collection methods adequate to address the research question? | Are the findings adequately derived from the data? | Is the interpretation of results sufficiently substantiated by data? | Is there coherence between qualitative data sources, collection, analysis, and interpretation? |
| Agha et al., (2021) | Yes | Yes | Yes | Yes | Yes | Yes | Yes |
| Dewi et al., (2021) | Yes | Yes | Yes | Yes | Yes | Yes | Yes |
| Ng et al., (2020) | Yes | Yes | Yes | Yes | Yes | Yes | Yes |
| Ng DY et al., (2021) | Yes | Yes | Yes | Yes | Yes | Yes | Yes |
| Saeed et al., (2021) | Yes | Yes | Yes | Yes | Yes | Yes | Yes |
| Hossaini et al., (2020) | Yes | Yes | Yes | Yes | Yes | Yes | Yes |
| Jenkins et al., (2020) | Yes | Yes | Yes | Yes | Yes | Yes | Yes |
| Lim et al., (2015) | Yes | Yes | Yes | Yes | Yes | Yes | Yes |
|  |  |  |  |  |  |  |  |
| Taib et al., (2014) |  |  |  |  |  |  |  |
| Norsa’adah et al, (2012) |  |  |  |  |  |  |  |
| Rastad et al., (2012) | Yes | Yes | Yes | Yes | Yes | Yes | Yes |
